# Comparison of Apixaban, Rivaroxaban, Dabigatran, and Vitamin K Antagonists in Patients With Atrial Fibrillation and Liver Disease: A Network Meta-Analysis

**DOI:** 10.7759/cureus.72351

**Published:** 2024-10-25

**Authors:** Sanam Shaikh, Gautham Varun Krishna Mohan, Bansari Patel, Sindhuja Sompalli, Ihtisham Habib, Sandipkumar S Chaudhari, Calvin R Wei, Areeba Khan

**Affiliations:** 1 Internal Medicine, Yangtze University, Jingzhou, CHN; 2 Internal Medicine, Tirunelveli Medical College, Tirunelveli, IND; 3 School of Medicine, American University of Barbados, Bridgetown, BRB; 4 Internal Medicine, Jagadguru Sri Shivarathreeshwara Medical College, Mysuru, IND; 5 Internal Medicine, Medical Teaching Institute, Lady Reading Hospital, Peshawar, PAK; 6 Cardiothoracic Surgery, University of Alabama at Birmingham, Birmingham, USA; 7 Family Medicine, University of North Dakota School of Medicine and Health Sciences, Fargo, USA; 8 Research and Development, Shing Huei Group, Taipei, TWN; 9 Critical Care Medicine, United Medical and Dental College, Karachi, PAK

**Keywords:** apixaban, atrial fibrillation, dabigatran, liver disease, rivaroxaban

## Abstract

Atrial fibrillation (AF) patients with liver disease present unique challenges in anticoagulation management due to increased risks of both thromboembolism and bleeding. This network meta-analysis aimed to compare the efficacy and safety of direct oral anticoagulants (DOACs), apixaban, rivaroxaban, and dabigatran, with vitamin K antagonists (VKAs) in this specific patient population. We conducted a comprehensive literature search across multiple databases, identifying seven studies (six observational and one randomized controlled trial) that met our inclusion criteria. The primary outcomes were the risk of stroke or systemic embolism (SE) and bleeding events.

Our analysis revealed that all three DOACs demonstrated superior efficacy and safety profiles compared to VKAs. Apixaban showed the most favorable outcomes, with the highest probability of being the most effective in preventing both stroke/SE (RR: 0.51, 95% CI: 0.38-0.67) and bleeding events (RR: 0.54, 95% CI: 0.43-0.69). Rivaroxaban and dabigatran also significantly reduced the risk of these outcomes compared to VKAs but to a lesser extent than apixaban. Notably, rivaroxaban was associated with a slightly increased bleeding risk compared to apixaban (RR: 0.76, 95% CI: 0.58-0.99). The consistency of our network model was confirmed through both global and local tests. While these findings provide valuable guidance for clinicians, the study's limitations, including the predominance of observational data, highlight the need for large-scale randomized controlled trials. Future research should focus on clearly defined anticoagulant dosing regimens and comprehensive assessments of cirrhosis status to further optimize anticoagulation strategies in AF patients with liver disease.

## Introduction and background

The most prevalent arrhythmia in adults is atrial fibrillation (AF), which affects millions of people globally [1]. Patients with AF are at a heightened risk of thromboembolic events due to blood flow pooling and stasis resulting from the disorganization of atrial contraction [2]. The management of AF, particularly the prevention of thromboembolic events, relies heavily on anticoagulation therapy. In addition to thromboembolic events, AF is associated with heart failure, stroke, cognitive decline, myocardial infarction, and impaired quality of life, often complicating management due to its chronic and progressive nature [3]. Vitamin K antagonists (VKAs), such as warfarin, have long been the standard of care for anticoagulation in AF [4]. However, their use is limited by a narrow therapeutic window, frequent monitoring, and numerous drug and dietary interactions. In recent years, direct oral anticoagulants (DOACs), including apixaban, rivaroxaban, and dabigatran, have emerged as alternatives to VKAs, offering more predictable pharmacokinetics, fewer interactions, and less frequent monitoring [5]. 

Liver disease adds another layer of complexity to anticoagulation therapy in patients with AF. Both liver disease and anticoagulants independently affect the coagulation system, either increasing the risk of thrombosis or bleeding [6]. In patients with liver disease, traditional VKAs are often problematic due to altered hepatic metabolism, impaired production of clotting factors, and fluctuating international normalized ratio (INR) levels, making anticoagulation management particularly challenging [7]. DOACs, with their different mechanisms of action and hepatic metabolism, offer potential advantages over VKAs in patients with liver disease [8]. However, the evidence regarding their safety and efficacy in this specific population is limited. While apixaban, rivaroxaban, and dabigatran have shown favorable profiles in the general AF population, the unique pharmacokinetic and pharmacodynamic changes in liver disease raise concerns about their use [9-10].

According to a previous meta-analysis, individuals with liver cirrhosis and AF who use DOACs instead of VKAs have a decreased risk of severe bleeding, stroke, or systemic embolism (SE) as well as thromboembolic events [11]. However, as far as our knowledge is concerned, no meta-analysis has been conducted before comparing the different DOACs in patients with liver disease. Secondly, there is a lack of studies directly comparing the efficacy and safety of different DOACs in patients with both liver disease and AF. This creates a significant gap in the literature, as these patients represent a high-risk group for both thromboembolic and bleeding events, and the optimal anticoagulation strategy remains unclear. By conducting this network meta-analysis, we aim to fill this gap and provide evidence-based recommendations for clinical practice. 

This network meta-analysis aims to compare the safety and efficacy of apixaban, rivaroxaban, and dabigatran with VKAs in patients with AF and liver disease. By pooling data from available studies, we aim to provide comprehensive insights into the optimal anticoagulation strategy for this complex patient population, considering both thromboembolic prevention and bleeding risk.

## Review

Methodology 

The Preferred Reporting Items for Systematic Reviews and Meta-Analyses (PRISMA) Extension Statement for Reporting Systematic Reviews Incorporating Network Meta-analyses of Health Care Interventions (PRISMA-NMA) was followed in the execution of this systematic review and network meta-analysis. 

Literature Search and Search Strategy 

A comprehensive literature search was conducted by two authors independently to identify relevant studies comparing apixaban, rivaroxaban, dabigatran, and vitamin K antagonists (VKAs) in patients with AF and liver disease. The search was performed across multiple databases, including PubMed, Excerpta Medica Database (Embase), and Web of Science, covering all publications up to September 10, 2024. We used a combination of MeSH terms and free-text keywords related to "atrial fibrillation", "liver disease", "direct oral anticoagulants", "DOACs", "apixaban", "rivaroxaban", "dabigatran", and "vitamin K antagonists". In addition, manual searches of the reference lists of key articles and relevant reviews were conducted to ensure no important studies were overlooked. Disagreements between authors were resolved through discussion and consensus. 

Study Selection 

The studies identified through the initial search were screened based on predefined inclusion and exclusion criteria. Eligible studies included randomized controlled trials (RCTs), cohort studies, and observational studies comparing the efficacy and safety of any of the DOACs (apixaban, rivaroxaban, and dabigatran) either with VKAs or other DOACs in patients with both AF and liver disease. Studies were excluded if they involved patients without liver disease, lacked a comparison group, or reported insufficient data for analysis. Two independent reviewers screened the titles and abstracts, followed by a full-text review of potentially eligible studies to confirm their inclusion. Disagreements between authors were resolved through discussion and consensus. 

Data Extraction 

Data extraction was performed independently by two reviewers using a standardized form and a third author cross-checked the data and entered it into Microsoft Excel (Microsoft Corp., Redmond, Washington, United States) for data analysis by importing it into R-studio (RStudio Team. (2015). RStudio: Integrated Development Environment for R. Boston, MA. Retrieved from http://www.rstudio.com/). The extracted data included study characteristics (author, year, study design, and sample size), interventions (type of anticoagulant and dosage), and relevant outcomes. The primary outcomes were the risk of stroke or SE and bleeding events. Discrepancies in data extraction were resolved through discussion or by consulting a third reviewer. 

Statistical Analysis 

Using a multivariate random-effects model built in the netmeta package of R statistical software version 4.0.2 (R Foundation for Statistical Computing, Vienna, Austria), the network meta-analyses were carried out within a frequentist framework. We added a continuity adjustment of 0.5 to each cell for all effect measures in order to handle sparse binary outcome data, which included some trials that recorded zero events in either group. Additionally, we calculated odds ratios using the Mantel-Haenszel method, which is particularly effective for evaluating binary outcomes, especially when data is limited. All statistical tests were two-sided, and a p-value of less than 0.05 was deemed statistically significant. We assessed the ranking probabilities of the various antiviral agents through their P-scores, which range from 0 (indicating the worst performance) to 1 (indicating the best). A higher P-score reflects superior overall efficacy among the competing treatments, and these P-score values are closely aligned with the area under the cumulative ranking curve. 

Results 

**Figure 1:**
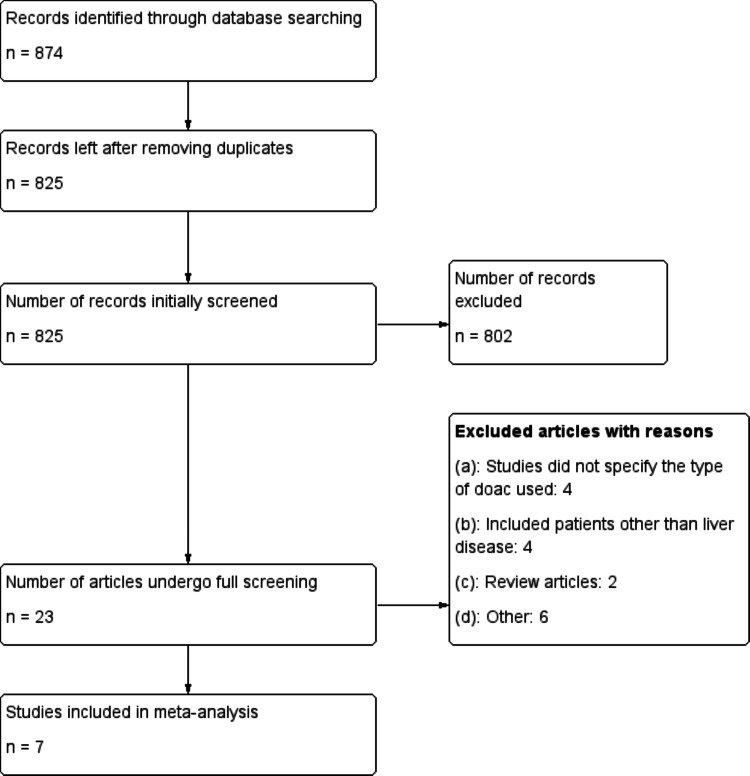
PRISMA flowchart of study selection PRISMA: Preferred Reporting Items for Systematic Reviews and Meta-Analyses; DOAC: Direct oral anticoagulant

Figure 1 illustrates the PRISMA flowchart for study selection. A total of 874 studies were identified through electronic database searches. After removing duplicates, 825 studies proceeded to the initial screening phase. Of these, 802 studies were excluded, and 23 articles underwent detailed evaluation based on the inclusion and exclusion criteria. Ultimately, seven studies were selected for inclusion in this meta-analysis with a pooled sample size of 64,593. The studies comprised four on dabigatran, five on apixaban, six on rivaroxaban, and six on VKAs. Table 1 summarizes the characteristics of the included studies, with six being observational and one an RCT. 

**Table 1:** Study characteristics (N=7) VKAs: Vitamin K antagonists; RCT: Randomized controlled trial; DOAC: Direct oral anticoagulant

Author ID	Design	Groups	Population	Follow-up	Dose of DOAC
Baylo et al., 2023 [12]	RCT	Dabigatran	30	Not reported	110 mg
		VKAs	26		
Douros et al., 2024 [13]	Observational	Apixaban	1696	Not reported	Not reported
		Rivaroxaban	1264		
		VKAs	1702		
Huang et al., 2024 [14]	Observational	Dabigatran	2011	10 Months	110 mg
		Rivaroxaban	2202		10-15 mg
Lawal et al., 2023 [15]	Observational	Apixaban	2721	6.84 Months	5 mg
		Rivaroxaban	2211		20 mg
		VKAs	4421		
Lee et al., 2019 [16]	Observational	Apixaban	171	15.6 Months	2.5 mg
		Rivaroxaban	732		10-15 mg
		Dabigatran	535		110 mg
		VKAs	946		
Lee et al., 2019 [17]	Observational	Apixaban	5561	14.4 Months	2.5 mg
		Rivaroxaban	10440		10-15 mg
		Dabigatran	6724		110 mg
		VKAs	12778		
Simon et al., 2024 [18]	Observational	Apixaban	2785	Not reported	Not reported
		Rivaroxaban	2785		
		VKAs	2852		

Network Consistency 

We evaluated the consistency between indirect and direct evidence for drug comparisons across both outcomes assessed in this study: risk of stroke or SE and bleeding events. Our assessment employed both global and local consistency approaches. For the stroke or SE outcome, the global consistency test yielded a p-value of 0.7615, supporting the null hypothesis of consistency and indicating the acceptability of the network model. Subsequently, we conducted local inconsistency tests for this outcome. All pairwise treatment comparisons resulted in statistically non-significant p-values, further corroborating the consistency of the model for the stroke or SE outcome. 

Similarly, we performed global and local inconsistency tests for bleeding outcomes. The global consistency test revealed no evidence of inconsistency (p-value: 0.5397). Local inconsistency tests for bleeding outcomes also yielded non-significant p-values across all comparisons, once again confirming the consistency between indirect and direct comparisons. These results collectively support the validity of our network meta-analysis model for both outcomes, enhancing the reliability of our findings. 

Comparison of Stroke or SE between Apixaban, Rivaroxaban, Dabigatran, and VKAs 

**Figure 2:**
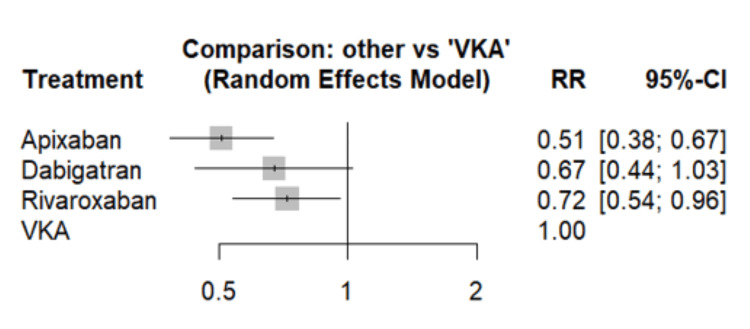
Comparison of risk of stroke or systemic embolism between different groups VKA: Vitamin K antagonists; RR: Risk ratio; CI: Confidence interval Adapted from [13,15-18]

**Figure 3:**
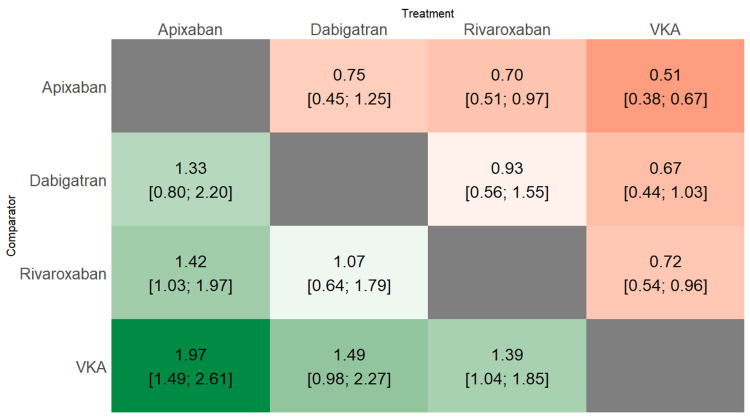
Heat plot comparing the risk of stroke or systemic embolism All values presented as RR (95% CI) VKA: Vitamin K antagonists; RR: Risk ratio; CI: Confidence interval Adapted from [13,15-18]

Figure 2 illustrates the comparison of stroke or SE risk between different DOACs and VKAs as the reference group. All three types of DOACs demonstrated a reduced risk of stroke or SE compared to VKAs. The difference between apixaban and VKAs was significant (RR: 0.51, 95% CI: 0.38-0.67), as was the difference between rivaroxaban and VKAs (RR: 0.72, 95% CI: 0.54-0.96). However, the difference between dabigatran and VKAs was not statistically significant (RR: 0.67, 95% CI: 0.44-1.03). Figure 3 presents a heat plot comparing all treatments with each other. Compared to apixaban, both rivaroxaban and dabigatran were associated with an increased risk of stroke or SE. We calculated P-scores for each treatment to assess their relative efficacy, with higher scores indicating better performance. The P-score of apixaban is highest among all treatments assessed (0.9487), followed by dabigatran (0.5700), rivaroxaban (0.4661), and VKAs (0.0151). These results suggest that among the treatments assessed, apixaban demonstrates the highest probability of being the most effective in preventing stroke or systemic embolism, while VKAs show the lowest. 

Comparison of Bleeding Events between Apixaban, Rivaroxaban, Dabigatran, and VKAs

**Figure 4:**
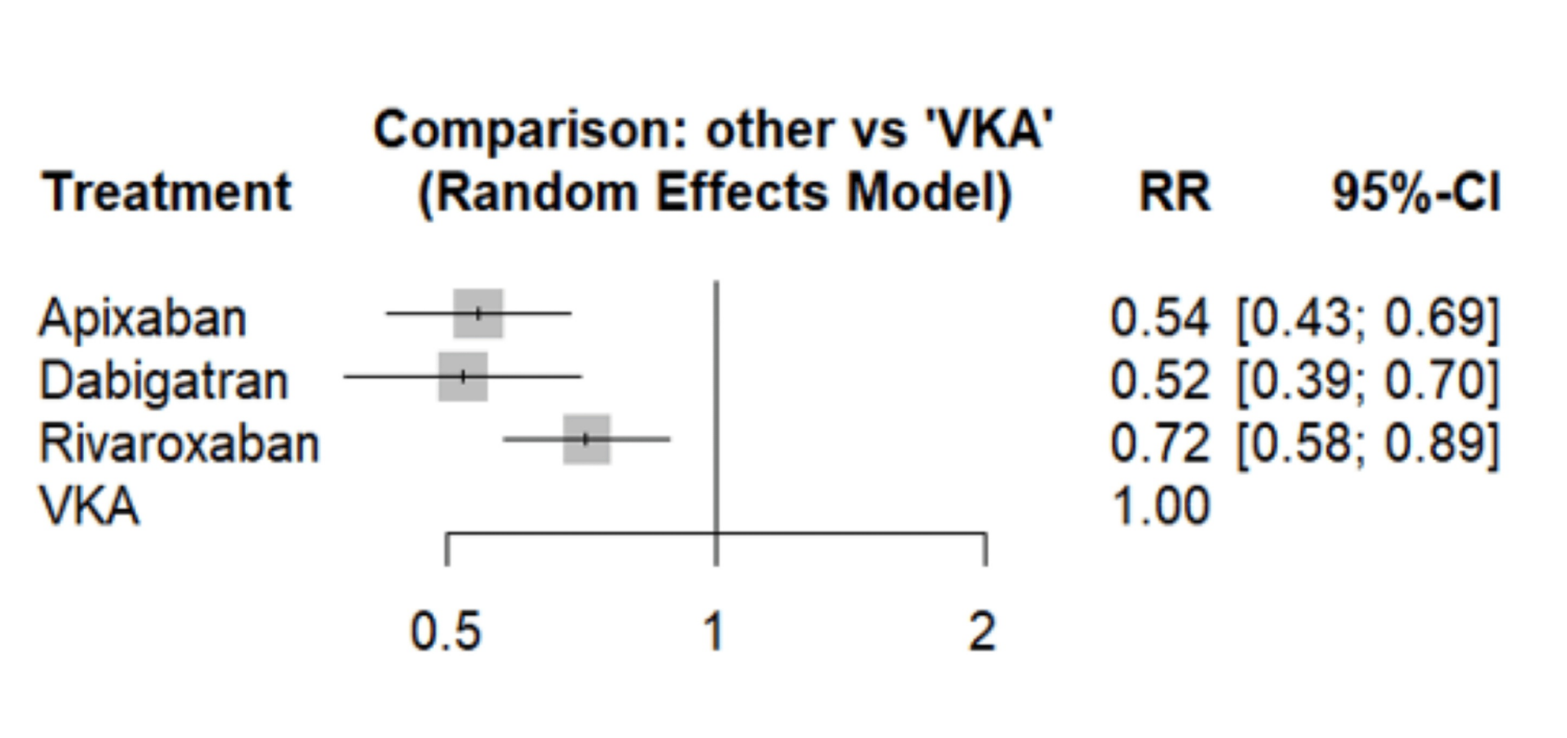
Comparison of the risk of bleeding events between different groups VKA: Vitamin K antagonists; RR: Risk ratio; CI: Confidence interval Adapted from [12-17]

**Figure 5:**
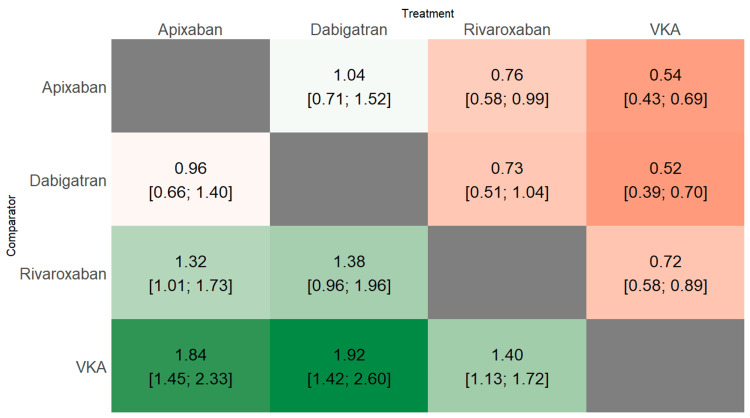
Heath plot comparing the risk of bleeding events All values presented as RR (95% CI) VKA: Vitamin K antagonists; RR: Risk ratio; CI: Confidence interval Adapted from [12-17]

In our analysis of bleeding event risks associated with various anticoagulants, DOACs demonstrated superior safety profiles compared to VKAs, as illustrated in Figure 4. When compared to VKAs, all three DOACs showed a statistically significant reduction in bleeding risk: apixaban (RR 0.54, 95% CI: 0.43-0.69), dabigatran (RR 0.52, 95% CI: 0.39-0.70), and rivaroxaban (RR 0.72, 95% CI: 0.58-0.89). The heat plot in Figure 5 provides a comprehensive comparison of all treatments, revealing that rivaroxaban is associated with a slightly increased risk of bleeding events compared to apixaban (RR: 0.76, 95% CI: 0.58-0.99). However, no significant differences in bleeding events were observed between dabigatran and apixaban, or between dabigatran and rivaroxaban. To further evaluate the relative efficacy of these treatments, we calculated P-scores, where higher scores indicated better performance in preventing bleeding events. Apixaban achieved the highest P-score (0.8547), followed by dabigatran (0.7975), rivaroxaban (0.7754), and VKA (0.003). These P-scores suggest that apixaban has the highest probability of being the most effective in preventing bleeding events, while VKAs demonstrate the lowest, aligning with the RR comparisons and providing a comprehensive view of the relative safety profiles of these anticoagulants. 

Discussion

In this network meta-analysis, we compared the safety and efficacy of apixaban, rivaroxaban, dabigatran, and VKAs in patients with AF and liver disease. Our results demonstrate that all three DOACs, apixaban, rivaroxaban, and dabigatran, offer significant advantages over VKAs in reducing the risk of both stroke or SE and major bleeding events. Among the DOACs, apixaban showed the most favorable outcomes, with the highest probability of being the most effective for stroke prevention and bleeding reduction. These findings align with previous studies in general AF populations but offer novel insights for the liver disease subgroup [19-20]. To the best of our knowledge, this is the first meta-analysis comparing apixaban, rivaroxaban, dabigatran, and VKAs in patients with AF and liver disease. 

The management of anticoagulation in AF patients with liver disease poses significant challenges due to the delicate balance between thrombosis and bleeding risks [21]. In our analysis, all DOACs demonstrated efficacy and safety, but apixaban emerged as the most favorable option. Our results, which show a comparable risk of ischemic stroke across the DOACs and lower bleeding risk with apixaban compared to rivaroxaban or dabigatran, align with findings from a recent systematic review and network meta-analysis of RCTs [22]. Similarly, Bai et al. found no significant difference between rivaroxaban and dabigatran in preventing thromboembolic events [23]. The increased bleeding risks associated with rivaroxaban compared to dabigatran or apixaban may be linked to their respective dosing regimens. While the plasma half-lives of the DOACs are comparable, rivaroxaban is administered once daily, whereas dabigatran and apixaban are dosed twice daily [24]. This once-daily dosing could potentially result in higher peak plasma concentrations, contributing to an elevated risk of bleeding. However, to date, no direct correlation has been established between rivaroxaban plasma levels and the incidence of bleeding events. 

In contrast to all DOACs, warfarin requires careful management to keep patients within a narrow therapeutic range. When levels fall below this range, warfarin loses its effectiveness in preventing strokes, and exceeding the range significantly heightens the risk of bleeding complications [25]. Additionally, the common issue of polypharmacy in patients with end-stage renal disease (ESRD) can further complicate the safety and efficacy of warfarin therapy [26]. 

All DOAC treatments should be approached with caution in patients with cirrhosis. Recent evidence has raised concerns regarding the potential for rivaroxaban to cause liver injury. Additionally, the FDA has indicated that rivaroxaban and edoxaban are contraindicated for patients with Child-Pugh class B cirrhosis. However, these findings have limitations. Much of the scientific data is derived from retrospective observational studies, as individuals with liver cirrhosis were often excluded from large randomized trials assessing the efficacy and safety of oral anticoagulants. Furthermore, the diagnosis of AF varied across studies, and the number of patients with liver cirrhosis included appears to represent only a subgroup of those with liver disease. Data on advanced liver disease (Child-Pugh class B or C) is scarce, even within these retrospective analyses, and the anticoagulant agents used varied from VKAs to different DOACs. Acknowledging the important link between liver and cardiovascular conditions underscores the necessity for large, randomized trials with clearly defined anticoagulant dosing regimens and comprehensive assessments of cirrhosis status to evaluate the safety and efficacy of these treatments in this population. 

Our network meta-analysis has systematically evaluated four AF treatments by several efficacy criteria. However, some limitations prevent us from further exploring the relative efficacy and safety. Firstly, only seven studies were included in this meta-analysis. Out of these seven studies, only one was an RCT. Due to the high number of observational studies, the present meta-analysis findings may be associated with selection bias that affects the generalizability of the findings. Secondly, there is a considerable variation in the number of studies conducted on various therapies, which could have a substantial effect on the final outcome. Lastly, the unevenness of analysis may result from variations in the methods used to collect the data, the inclusion and exclusion criteria, and the standards used to define the outcomes. It is important to perform large-scale RCTs to understand how different DOACs affect the outcomes in liver disease patients in order to guide clinicians in prescribing drugs promoting good prognoses. 

## Conclusions

In conclusion, this network meta-analysis provides valuable insights into the efficacy and safety of DOACs compared to VKAs in patients with AF and liver disease. All three DOACs demonstrated superior outcomes to VKAs, with apixaban emerging as the most favorable option for both stroke prevention and bleeding risk reduction. These findings offer important guidance for clinicians managing this complex patient population. However, the study's limitations, including the predominance of observational studies and potential selection bias, underscore the need for large-scale RCTs. Future research should focus on clearly defined anticoagulant dosing regimens and comprehensive assessments of cirrhosis status to further evaluate the safety and efficacy of these treatments. As the link between liver and cardiovascular conditions becomes increasingly apparent, continued investigation in this area is crucial for optimizing patient care and outcomes.

## References

[1] Benjamin EJ, Muntner P, Alonso A (2019). Heart disease and stroke statistics-2019 update: a report from the American Heart Association. Circulation.

[2] Go AS, Fang MC, Udaltsova N, Chang Y, Pomernacki NK, Borowsky L, Singer DE (2009). Impact of proteinuria and glomerular filtration rate on risk of thromboembolism in atrial fibrillation: the Anticoagulation and Risk Factors in Atrial Fibrillation (ATRIA) study. Circulation.

[3] Lucà F, Giubilato S, Di Fusco SA (2021). Anticoagulation in atrial fibrillation cardioversion: what is crucial to take into account. J Clin Med.

[4] Kirchhof P, Benussi S, Kotecha D (2016). 2016 ESC guidelines for the management of atrial fibrillation developed in collaboration with EACTS. Eur J Cardiothorac Surg.

[5] Levy JH, Spyropoulos AC, Samama CM, Douketis J (2014). Direct oral anticoagulants: new drugs and new concepts. JACC Cardiovasc Interv.

[6] Karapedi E, Papadopoulos N, Trifylli EM, Koustas E, Deutsch M, Aloizos G (2022). Anticoagulation in patients with atrial fibrillation and liver cirrhosis. Ann Gastroenterol.

[7] Intagliata NM, Davis JP, Caldwell SH (2018). Coagulation pathways, hemostasis, and thrombosis in liver failure. Semin Respir Crit Care Med.

[8] Costache RS, Dragomirică AS, Gheorghe BE, Balaban VD, Stanciu SM, Jinga M, Costache DO (2023). Oral anticoagulation in patients with chronic liver disease. Medicina (Kaunas).

[9] Giorgi MA, Cohen Arazi H, Gonzalez CD, Di Girolamo G (2011). Changing anticoagulant paradigms for atrial fibrillation: dabigatran, apixaban and rivaroxaban. Expert Opin Pharmacother.

[10] Menichelli D, Ronca V, Di Rocco A, Pignatelli P, Marco Podda G (2021). Direct oral anticoagulants and advanced liver disease: a systematic review and meta-analysis. Eur J Clin Invest.

[11] Sinha T, Kaur M, Mayow AH (2024). Effectiveness of direct oral anticoagulants and vitamin K antagonists in preventing stroke in patients with atrial fibrillation and liver cirrhosis: a systematic review and meta-analysis. Cureus.

[12] Baylo A, Cherniavskyi V, Reshotko D (2023). Assessment of the efficiency and safety of anti-coagulation therapy in patients with liver cirrhosis and atrial fibrillation. Clin Exp Hepatol.

[13] Douros A, Cui Y, Platt RW, Filion KB, Sebastiani G, Renoux C (2024). Effectiveness and safety of direct oral anticoagulants among patients with non-valvular atrial fibrillation and liver disease: a multinational cohort study. Thromb Res.

[14] Huang X, Xu W, Wu G (2024). Efficacy and safety of dabigatran and rivaroxaban in atrial fibrillation patients with impaired liver function: a multicenter retrospective cohort study. Eur J Clin Pharmacol.

[15] Lawal OD, Aronow HD, Shobayo F (2023). Comparative effectiveness and safety of direct oral anticoagulants and warfarin in patients with atrial fibrillation and chronic liver disease: a nationwide cohort study. Circulation.

[16] Lee HF, Chan YH, Chang SH (2019). Effectiveness and safety of non-vitamin K antagonist oral anticoagulant and warfarin in cirrhotic patients with nonvalvular atrial fibrillation. J Am Heart Assoc.

[17] Lee SR, Lee HJ, Choi EK (2019). Direct oral anticoagulants in patients with atrial fibrillation and liver disease. J Am Coll Cardiol.

[18] Simon TG, Singer DE, Zhang Y, Mastrorilli JM, Cervone A, DiCesare E, Lin KJ (2024). Comparative effectiveness and safety of apixaban, rivaroxaban, and warfarin in patients with cirrhosis and atrial fibrillation: a nationwide cohort study. Ann Intern Med.

[19] Zeng S, Zheng Y, Jiang J, Ma J, Zhu W, Cai X (2022). Effectiveness and safety of DOACs vs. warfarin in patients with atrial fibrillation and frailty: a systematic review and meta-analysis. Front Cardiovasc Med.

[20] Li Y, Wu S, Zhou J, Zhang J (2024). Efficacy and safety of direct oral anticoagulants in patients with atrial fibrillation combined with chronic kidney disease: a systematic review and meta-analysis. Thromb J.

[21] Chang WH, Mueller SH, Tan YY, Lai AG (2021). Antithrombotic therapy in patients with liver disease: population-based insights on variations in prescribing trends, adherence, persistence and impact on stroke and bleeding. Lancet Reg Health Eur.

[22] Cohen AT, Hill NR, Luo X, Masseria C, Abariga SA, Ashaye AO (2018). A systematic review of network meta-analyses among patients with nonvalvular atrial fibrillation: a comparison of efficacy and safety following treatment with direct oral anticoagulants. Int J Cardiol.

[23] Bai Y, Deng H, Shantsila A, Lip GY (2017). Rivaroxaban versus dabigatran or warfarin in real-world studies of stroke prevention in atrial fibrillation: systematic review and meta-analysis. Stroke.

[24] Ageno W, Beyer-Westendorf J, Rubboli A (2017). Once- versus twice-daily direct oral anticoagulants in non-valvular atrial fibrillation. Expert Opin Pharmacother.

[25] Szummer K, Gasparini A, Eliasson S (2017). Time in therapeutic range and outcomes after warfarin initiation in newly diagnosed atrial fibrillation patients with renal dysfunction. J Am Heart Assoc.

[26] AlTurki A, Marafi M, Dawas A (2024). Meta-analysis evaluating apixaban in patients with atrial fibrillation and end-stage renal disease requiring dialysis. J Arrhythm.

